# Factors associated with health-related quality of life among Indian women in mining and agriculture

**DOI:** 10.1186/1477-7525-11-9

**Published:** 2013-01-22

**Authors:** Melba Sheila D’Souza, Subrahmanya Nairy Karkada, Ganesha Somayaji

**Affiliations:** 1 Department of Adult Health and Critical Care, College of Nursing, Sultan Qaboos University, GSM, Al-Khoud, PO 66, Muscat 123, Sultanate of Oman; 2 Department of Business Studies, Higher College of Technology, Al Khuwair, Ministry of Manpower, Muscat, Sultanate of Oman; 3 Department of Sociology, Goa University, TaleigaoPlatuea, Goa, India

**Keywords:** SF-36, Health-related quality of life, Well-being, Perceived health, Women’s health, Coping, Nursing, Mining

## Abstract

**Background:**

Women facing social and economic disadvantage in stressed communities of developing countries are at greater risk due to health problems. This paper investigates the relationships between structural, health and psychosocial predictors among women in mining and agricultural communities. This paper is a report of a study of the predictors of the health-related quality of life among Indian women in mining and agricultural communities.

**Methods:**

A descriptive cross-sectional research design was used. The instruments used are SF-36 Health Survey and Coping Strategy Checklist. ANOVA, MANOVA and GLM were used in the analysis. The study was conducted between January-September 2008 with randomly selected women in a mining (145) and an agricultural community (133) in India.

**Results:**

Women in the agricultural community had significantly increased Physical Health, Mental Health and SF36 scores compared with those in the mining community. Years of stay, education and employment were significant predictors among women in the agricultural community. 39% (33%) and 40% (26%) of the variance in Physical and Mental health respectively among women in agricultural and mining communities are predicted by the structural, health and psychosocial variables.

**Conclusion:**

Perceived health status should be recognised as an important assessment of Physical and Mental Health among women in rural stressed communities. Cognitive, emotional and behavioural coping strategies are significant predictors of health related quality of life. Implications. Nurses should use the SF-36 as a diagnostic tool for assessing health related quality of life among women and discuss coping strategies, so that these can target women’s adaptive behaviour. This should be an essential part of the nursing process for facilitating adaptive process for improved health related quality of life.

## Background

Mining in India is an important developmental activity and has a manifold impact on population dynamics, development and environment. Men and women are differentially exposed or vulnerable to determinants of health [1,2]. Vulnerable groups, especially women, the elderly and children, often live in poorer socio-economic conditions and have poorer literacy skills compared with their urban counterparts [3]. Rural health status in stressed communities (e.g. mining) is lower than for non-stressed communities (e.g. agricultural) and limited access to health and welfare support services can further jeopardise the health status of rural women in India [4]. Studies also show that women’s interaction with the biophysical environment within their own ‘life spaces’ reveals that they are exposed to the hazards of environmental illness in a manner that is clearly environmentally-differentiated (socio-economic, cultural and biophysical) from less stressed communities [5-9].

Health-related quality of life (HRQOL) is a perception of the degree of contentment with and capability to perform and control different facets of one’s life [10-12]. For the purpose of our study HRQOL is defined as the perceived health status and daily functioning including physical and mental health, role limitations, and social functioning. Coping with stimuli is an important indicator of positive health status and is linked to health-related quality of life [13]. Coping strategies refer to behaviours adopted by women during stressful situations [14,15]. Coping is conceptualized as being an important personality variable representing cognitive, behavioural and emotional coping strategies targeted at modifying the cause of a stressful problem. Women differentially select particular coping strategies across stressful events [16,17]. This empirical research is a study of women’s perceived health that could possibly be exerted by the structural (demographic, socio-economic), health (cultural, illness) and psychosocial (support, coping) factors contingent in mining and agricultural communities.

Living in mining communities exposes disadvantaged groups to health problems related to contamination of air, water, food, soil and river beds with toxic chemical and metallic discharges [18]. Women employed in the mines or collieries are exposed to toxic and hazardous substances due to poor safety, lack of control and monitoring measures and, as a result, and are susceptible to risks from several occupational illnesses [19]. Women living near coal and uranium mining in Bihar have succumbed to illness like malaria, typhoid and hepatitis, which were not prevalent before the mining activities. High infant mortality and deteriorating health among women were also common [20]. Women living near the Kolar gold fields travel far for meager wages to support their families, while most of the men are sick, depressed and unemployed due to the closure of the mines or have died in occupational accidents [20,21]. Most of the rural/tribal women due to mining-induced exploitation suffer from the social and particularly ill effects of mining-related hazards, pollution, poor waste disposal, denial of access to natural resources (water, forest, agriculture, land), incomplete rehabilitation, poor housing facilities, mine disasters, lack of livelihood, accidents, chemical spillage and closure [22,23]. These effects culminate in poor health and well-being among women depending on the thresholds of exposure in the mining communities compared to the less stressed communities.

### Review of literature

Women face torture from security guards as they collect mud from the tailings to retrieve minute specks of gold. Unemployment has also led to the growth of anti-social activities like theft, fights and murder causing insecurity to women [20,21]. Incidences of alcoholism, drug addiction, prostitution, gambling, incest, wife swapping, infidelity, and domestic violence against women, have resulted in an increased risk of AIDS and STD among women living in mining areas [18]. The problems of the adivasi (local tribe) women due to the mining induced exploitation are poor quality of drinking water, roads, housing, agriculture produce, wife-battering, alcoholism, desertion, unemployment, starvation, gambling, infidelity, and AIDS [22].

In the coal mines of Hazarbagh, women are often scared, harassed and assaulted by the “coal mafia” [24]. Mining activities tend to reduce opportunities for women in Orrisa, thus increasing their levels of dependency and vulnerability.

AIDS have a specific impact on women in the mining town of Timika (West Papua, Indonesia) due to increasingly high levels of alcohol-related violence, infidelity, rape and prostitution. Most of the women in the mining areas are malnourished, prone to AIDS/STD infections, family violence, rape and prostitution, fueled by alcohol abuse [25]. The local women in the Soroako gold mining areas (Sulawesi, Indonesia) received welfare benefits like food packages after delivery, literacy classes, and medical services. There were also growing incidence of teenage pregnancy, alcoholism, rape and other forms of violence against women [26].

Hence stress and stressful life events are related to a number of disorders, both psychological and physical, among women [27,28]. Effective coping strategies such as support from social networks, and spirituality, can reduce stress, promote physical and mental wellbeing, and improve health. There is no review of literature regarding domestic violence, cultural beliefs, physical or mental health status and coping among women in the local mining community. A few local studies record high air, water and noise pollution, dumps, silt and loss of agricultural fields and poor quality of life among communities living in the mining regions [29-31]. This paper focuses on perceived women’s health, use of coping strategies and health related quality of life. This is the first paper highlighting perceived women’s health, coping and predictors of health-related quality of life in the mining villages compared to a non-stressed economic community (i.e. agricultural village).

### Conceptual framework

**Figure 1:**
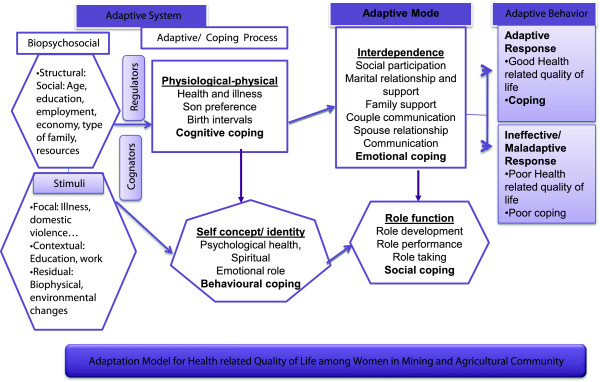
Adaptation model for health related quality of life among women in mining and agricultural community.

Roy’s adaptation model views the biopsychosocial individual as an adaptive system that must adapt to environmental stimuli [32]. Adaptation is considered to take place in one biological and three psychosocial modes as responses to the environment and to a particular situation which entail a great deal of motivation from the one involved [33]. In our study, a women’s adaptive system (structural, health and psychosocial) coupled with coping processes through cognator and regulator subsystems act to maintain adaptation in the four adaptive modes: physiologic-physical, self-concept-group identity, role function, and interdependence (Figure 1).

We assessed the women’s self-perceived health (dimensions) in each of the adaptive modes and the relevant internal and external stimuli (focal, contextual, and residual) influencing those adaptive abilities in the mining and agricultural communities (Appendix A). Relationships between the adaptive modes occurs when stimuli affect more than one mode and coping processes are used to expand those abilities and to enhance environmental interactions to attain the goal, i.e., health status (adaptation). This identification leads to goal setting to promote adaptation in the four adaptive modes to enhance women’s interaction with her immediate environment. Women’s adaptive responses occur through use of adaptive process or coping strategies (cognitive, social, emotional, and behavioural) that help her integrate mutually with the environment resulting in adaptation. When all sub systems function well, adaptive behaviour (health related quality of life) occur, otherwise maladaptation (ineffective response) occurs. Nursing interventions should focus on altering these stimuli or strengthening adaptive processes to result in adaptive behaviours.

## Methods

### Aim

The aim of the study was to examine the relationship between health-related quality of life and possible predictors of HRQOL among Indian women in mining and agricultural communities.

### Design

A descriptive cross-sectional research design was selected for the study.

### Settings and sampling

**Figure 2:**
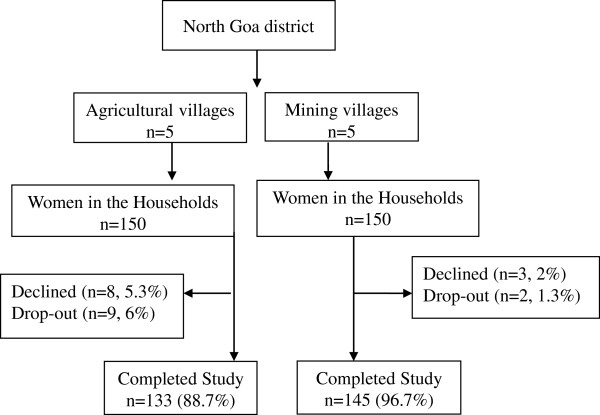
Multi-stage sampling strategy using simple random sampling.

This study has been conducted in the active iron ore mining villages in Goa which has a high air pollution index in 2008 [29,30]. A two-stage random sample was obtained from census tracts, electoral lists and family list from the Directorate of Health Services, local *panchayats* and the primary health centres respectively from the villages in the mining and agriculture communities (Figure 2).

The sample size was initially determined using G*Power software with the intention of using a linear multiple regression analysis [34,35] for the mining community. The sample size was calculated at a power of 0.9 with an effect size of 0.15 using 10 predictors (independent variables) with an alpha of 0.05. The effect size was computed assuming a correlation coefficient of 0.3–0.4 between the number of years living in a mining community (metric/variable) with Physical and Mental Health. It was found that a minimum sample size of 147 would be needed for these input parameters (rounded to 150). A matching sample size of 150 was decided for the agricultural community,

A multi-stage simple random sampling was used to select the women for the study (Figure 2). In the first stage in the North Goa district, Goa, five mining and five agricultural villages were selected randomly from the census tract from the Directorate of Health Services. In each selected village, 150 households were selected randomly from the electoral list from the local panchayats in both the communities. From the family registration list was obtained from the primary health centre, one woman was selected randomly from each household to make a total of 150 women in the mining and agricultural communities respectively. Women who met the eligibility criteria were included in the study. Five women dropped out of the study, so the actual sample size was reduced to 145 women in the mining community. Among the 150 randomly selected households in the agricultural community, 8 women declined to participate and 9 women dropped out due to time and personal constraints, making it 133.

The inclusion criteria included local married women in the reproductive age group of 15–45 years who had borne at least one child, and women who are able to understand, converse, and communicate in Konkani and who would provide voluntary participation. We excluded women working in the mines.

### Data collection procedures

The investigators reviewed validated and standardized tools on health status and coping. One standardized tool was selected to achieve the objectives of the study based on the background of the women.

#### The SF-36 health survey (SF36)

The SF-36 Health Survey (Version 1.0) also known as the Medical Outcomes Study, has been adapted for this study population after carefully considering the items and adaptation process [36-42]. It is a self-administered questionnaire of 36 items with closed-ended, structured questions and has 8 multi-item scales, which concentrate on the respondents’ experiences, feelings, beliefs, perceptions and convictions concerning their health-related quality of life during the past four weeks [43-46]. The SF-36 Health Survey items and scales were constructed using the Likert scale method of summated ratings (Table 1). The first five scales make up the “Physical Health” or functional status dimension (subscale 1–5) , and the last five form the “Mental Health” or well-being dimension (subscale 4–7) . Hence, each dimension includes three specific and two overlapping (Vitality and General Health) scales. The SF-36 is divided into two aggregate summary measures, the Physical Component Summary or Physical Health (PCS or PH) and the Mental Component Summary or Mental Health (MCS or PH).

**Table 1:** SF-36 health survey consisting of eight health concept/scales/dimensions

**Concepts**	**SF 36 Health Dimensions**	**Total items**
	**Functional status subscales: 1,2, 6,7**	
1. Physical functioning (PF)	Perceptions of quality of life that are influenced by their physical condition. Performing vigorous activities, moderate activities or all types of physical activities including the most vigorous without limitation as a result of health.	10
2. Physical role limitations (RP)	Performance of the roles in daily activities is impeded by their physical state of health, e.g. ability to perform vigorous activities or moderate activities.	4
	Physical limitations in performance of daily living. Problems encountered with daily activities or work as a result of physical health or No problems with work or other daily activities as a result of physical health.	
6. Social functioning (SF)	Social activities and interaction with significant others such as family members, friends, neighbours and other social relations. Performance of social activities in lieu of health problem (eg, osteoarthritis) or normal social activities without interference.	2
7. Emotional – Role limitations (RE)	Emotional condition, e.g. feeling depressed or anxious, limits the daily functioning and ability to perform roles, such as in cutting down on the amount of time spent on work or other activities and accomplishing less than he/she would like to. Problems encountered with daily activities or work as a result of emotional health or No problems with work or other daily activities as a result of emotional problems.	3
	**Wellbeing subscales: 3-5,8**	
3. Bodily pain (BP)	The scores on this dimension indicate to what extent the respondents’ experience of bodily pain hinders their performance of daily activities, including work-related duties in the public domain and tasks within the home environment. Overall pain severity. No pain or limitations resulting from pain.	2
5. Vitality/energy/fatigue (VT)	Feeling energetic and full of pep, or worn out and tired.	4
	Frequency of feeling full of energy vs tired. Feels full of pep and energy all of the time.	
8. General Mental health (MH)	Psychological distress and psychological wellbeing dimension of the respondent is measured in terms of the extent to which he/she is inter alia feeling full of pep, is happy, is feeling calm and peaceful, is very nervous, or is feeling worn out and tired. Degree of nervousness or depression. Feels peaceful, happy	5
	**Overall evaluation of health subscale: 4**	
4. General health perceptions (GH)	Concepts such as excellent, very good, good, fair or poor, getting ill easier than other people, and just as healthy as anyone he/she knows. Overall general health. Evaluates personal health as excellent.	5
	**Total SF36 Health Survey scale**	35
**36th question**	“Compare health now with that one year ago?”, is not included within these eight scales	

Note. Physical health: subscales 1-5, Mental health: subscales 4-8.

The SF36 dimensions consist of: Physical functioning (PF, a pure physical health): 3–12 (10 items), Physical role limitations (RP): 13–16 (4 items), Bodily pain (BP): 21–22 (2 items), Vitality/energy/fatigue (VT): 23, 27, 29, 31 (4 items), General health (GH): 1, 33–36 (5 items), Mental health (MH, a pure mental health): 24–26, 28, 30 (5 items), Social functioning (SF): 32, 20 (2 items) and Emotional role limitations (RE): 17–19 (3 items). The scores of the two dimensions and the total SF36 score are based on mathematical averaging of the scale components (summated ratings) which assume that items within a hypothesized scale can be summed up without score standardization or item weighting [47]. A higher score indicates better level of HRQOL.

The SF-36® Health Survey items and scales were constructed using the Likert method of summated ratings. Answers to each question are scored (some items need to be recoded). These scores are then coded and summed to produce raw scale scores for each health concept which are then reweighted/transformed to a 0 (worst health) – 100 (best health) scale. Each scale score ranges from 0 to 100, with a higher score indicating a better level of QOL. The SF-36 can also be divided into two aggregate summary measures the Physical Component Summary (PCS) and the Mental Component Summary (MCS). Scoring algorithms can then be applied to produce the PCS and MCS scores. (These two summary scores have the major advantage of being norm based. They also have reduced floor and ceiling effects.)

#### Coping strategy checklist

The investigators reviewed the Coping Orientation for Problem Experiences (COPE) Inventory [16], Ways of Coping Questionnaire [48,49] and the Revised Ways of Coping Checklist [50] and Reproductive health semi-structured interview [51]. Based on these tools the investigators developed the Coping Strategy Checklist consisting of 25 items across four dimensions: Cognitive coping ([10] items): focused thinking, problem solving, reflection, belief/faith, identifying cause and consequences, anticipate, planning and prepare, systematic thinking, take precautions and relax. Behavioural coping ([5] items): use of health seeking behaviours, seeking medical treatment, spiritual help, assistance during illness and seeking help. Social coping ([4]) items: social participation, self-support groups, husband’s support, family support. Emotional coping ([6] items): communication, wishful thinking, self-blame, avoidance and suppression. Example ‘I talk to my husband to find out more about the situation’, ‘I talk to my mother who could do something concrete about the problem’.

#### Social and health status

This demographic or socio-economic status tool (15 items) include age, years of stay, family type, socio-economic status, education, employment status, family economic status, house ownership and health problems of women.

### Ethical considerations

Institutional Ethics Committee formed by Goa University approved the study and provided human and ethical clearance [52]. During the household visits the researchers interacted with the household members, built rapport with the family members, and sought participation among women and their spouses. This helped to gain co-operation with the women and their spouses during the data collection period in the area. In the mining and agricultural villages, 150 women selected randomly in each *waddo* (unit in each of the randomly selected village) were informed of the study by the research assistants through the primary health centres (Figure 2). They visited each woman participant in their houses to ascertain their participation, to develop a rapport and trusting relationship.

The purpose, procedures and ethical concerns of the study were informed to the local women. An introductory letter explaining the kind of research and the questionnaires was given to each woman randomly selected with the inclusion criteria from mining and agricultural villages. A verbal and a written informed consent document were obtained from the women indicating their voluntary participation and prerogative to withdrawal at any time. All women were assured that participation was voluntary, their anonymity, confidentiality of their responses, a blind analysis and a genuine use of the data would be adhered to. All the respondents were interviewed by the trained research assistants using the Konkani version of the tools. The women were interviewed in the privacy of their homes for 2 hours during convenient times from March-July 2008. Women were informed that the results would be reported accurately and that all shortcomings in the research, such as errors and limitations, would be disclosed [53-55]. Data were collected by trained interviewers directly from the selected women using pre-coded questionnaires. After the data collection the women were appreciated for their time and participation. All the tools were stored in locked cabinets with access to the researcher only.

### Data analysis

Data quality was satisfactory, with a high response rate and use of all response categories, suggesting that there were no problems related to the translation of items and categories in the Surveys. Using Microsoft Excel 97, Version 9.0 (Microsoft, Redmond, WA), we used a method for the SF36 scoring of the subscales/dimensions and the total SF36 [56], multivariate analysis of variance and General Linear Model [57-59]. For trustworthiness and validation of the appropriateness of the survey tools, some women were interviewed after the data analysis to discuss the results and findings of the study. The data interpretation was according to the women’s perception and experiences.

#### Validity and reliability

The SF36 Health Survey, Coping Strategy Checklist and the Social Health Status (English and *Konkani* versions) were validated with the content blueprint and three-point evaluation criteria. These tools were adapted by forward and backward translation, iterative revision, and consensus by experts. Each translation was reviewed by three independent health-care providers, psychologists and sociologists who were proficient in both English and Konkani. The accuracy of the translations was certified. There was 100% strong agreement about the items in each domain among these subject experts.

The SF-36 was administered to 20 randomly selected women in both the mining and the agricultural communities in early January 2008 for pre-testing and a try-out. All participating women were able to answer the questionnaires independently within 90 minutes. The tools were found to be culturally sensitive, clear, relevant, and did not have any problems with its format or any aspects of the SF36 Health, Coping Strategy Scale and Social and Health Surveys.

The internal consistency of items was evaluated by analysis of correlations between the items and their respective scales. The internal consistency of the scales was calculated using Cronbach’s alpha α coefficient. It is a measure of the extent to which items within the same scale correlate with each other. The α coefficient ranges from 0 to 1: values greater than 0.70 are considered acceptable for group comparisons, and 0.90 for person-level comparisons [40]. The response rate for the scales was 100%, i.e., all questions were answered by all women. Internal consistency reliability was high across most of the SF-36 subscales (> 0.70) indicating a high level of correlation amongst items within the same subscale. The overall Cronbach’s α coefficient of the SF-36 questionnaire was 0.76. Results showed that the SF-36 questionnaire demonstrated good reliability.

A pilot study was conducted in the February 2008 to assess the feasibility of the study, plan for the analyses and to determine the flaws in the design, using a random sampling technique with ten selected married women in the mining and agricultural communities. The pilot study did not show any problems or flaws in the design of the study.

## Results

### Social health status

A half-percentage of the women had a secondary education (53.4% and 40%), while a quarter percentage of the women were illiterate (23.3% and 24.8%) in the agricultural and mining communities respectively (Table 2). Only a few women were working after marriage (13.8% and 46.6%) compared with those before marriage (35.2% and 44.3%) in the mining community compared with the agricultural community. A substantial portion of the women had good (62.1% and 36.1%) economic status in the mining community compared with the agricultural community respectively.

**Table 2:** Socio-economic status among women in mining and agricultural communities

**Socio-Economic Status**		**Mining community**		**Agricultural community**	
		**N = 145**		**N = 133**	
Variable	Category	F	%	F	%
Age	Less than 30 years	36	24.8	37	27.8
	30-39 years	64	44.1	60	45.1
	More than 40 years	45	31.0	36	27.1
Marital status	Living with husband	139	95.9	127	95.5
	Separated/divorced	6	4.1	6	4.5
Years of stay	Less than 10 years	53	36.6	45	33.8
	10-20 years	33	22.8	48	36.1
	More than 20 years	59	40.7	40	30.1
Family type	Joint	24	16.6	20	15.0
	Nuclear	121	83.5	115	85.0
Educational status	No schooling	36	24.8	31	23.3
	Elementary (till 4th)	33	22.8	22	16.5
	Secondary (till 10th)	58	40.0	71	53.4
	Vocational courses	18	12.4	9	6.8
Employment before marriage	Farming	9	6.2	51	38.3
	Unskilled	42	29.0	8	6.0
Employment after marriage	Farming	5	3.4	48	36.1
	Unskilled	15	10.4	14	10.5
	Housewife	125	86.2	71	58.4
Economic status	Good	90	62.1	48	36.1
	Moderate	37	25.5	51	38.3
	Poor	18	12.4	34	25.6
Domestic violence	Wife beating	28	19.3	22	16.5
	No assault	117	80.7	98	73.7
Emotional coping	Present	42	29.0	71	53.4
	Absent	103	71.0	62	46.6
Cognitive coping	Present	29	20.0	80	60.2
	Absent	116	80.0	53	39.8
Behavioural coping	Present	38	26.2	41	31.1
	Absent	107	73.8	91	68.9
Social coping	Present	101	69.7	107	80.5
	Absent	44	30.3	26	19.5
Ownership	Own house	103	71.0	119	89.5
	Rental house	42	29.0	14	10.5
	Agricultural land	29	20.0	59	44.0

More women in the mining community reported domestic violence (19.3% and 16.5%) compared to the mining community. A greater percentage of women in the agricultural community used social (80.5% and 69.7%), cognitive (60.2% and 20%), emotional (53.4% and 29%) and behavioral (31.1% and 26.2%) coping strategies compared with to the women in the mining community respectively.

The women in the mining community reported more eye (54.48% and 42.86%), upper respiratory (30.34% and 24.06%), lower respiratory (49% and 19.55%) and psychosomatic (52.41% and 35.34%) illnesses compared to those in the agricultural community (Table 3). Women in the agricultural community (58.65%) reported more musculoskeletal illness than those in the mining community (48.28%).

**Table 3:** Enduring illness among women in the mining and agricultural communities

**Physical and Mental Health**	**Mining community**		**Agricultural community**	
	**N = 145**		**N =133**	
	**Freq**	**%**	**Freq**	**%**
Eye (inflammation, allergy, cataract)	79	54.48	57	42.86
Ear (infection, hearing deficit, inflammation)	16	11.03	18	13.53
Throat (inflammation, allergy, sore throat)	11	7.59	16	12.03
Skin (infection, boils, allergy)	14	9.66	11	8.27
Upper respiratory (common cold, sinus, dust allergy, dry cough)	44	30.34	32	24.06
Lower respiratory (cough with phlegm, congestion, infection)	49	33.79	26	19.55
Heart (chest pain, high BP, heart diseases)	15	10.34	17	12.78
Musculoskeletal (severe backache, joint pain, neck pain, injury, foot problems)	70	48.28	78	58.65
Digestive (severe backache, joint pain, neck pain, injury, foot problems)	15	10.34	19	14.29
Endocrine (diabetes, thyroid)	4	2.76	1	0.75
Psychological (body ache, headache, fatigue, lethargy, reduced sleep, insomnia, loss of sensation, dizziness, fainting, anxiety, worry)	76	52.41	47	35.34

### Dimensions of the SF36

To examine the mean values of the 8 dimensions of self-perceived health (HRQOL) among women in the mining and agricultural communities, ANOVA were used (Table 3).

H01 There is no significant difference between the SF36 mean scores among women in the mining and agricultural communities.

Among the 8 scales, women reported significantly higher scores on Physical Role Limitations (mean = 79.9 and 57.8, p < 0.05) and Emotional Role Limitations (mean = 78.7 and 73.1) in agricultural and mining communities (Table 4). Significantly higher differences were found in General Mental Health (mean = 69.8 and 63.6, p < 0.05) and Social Functioning (mean = 72.7 and 65.9, p < 0.01) among women in the agricultural and mining communities. Higher mean scores were observed for Physical Role Limitations (mean = 79.9), Emotional–Role Limitations (mean = 78.7), Bodily Pain (mean = 73.2), Social Functioning (mean = 72.7), General Mental Health (mean = 69.8), Vitality (mean = 61.8), General Health Perceptions (mean = 59.6), and Physical Functioning (mean = 53.7) among women in agricultural compared with women in the mining community. Women in the agricultural community achieved a significantly higher SF36 (mean = 68.7 and 62.7, p < 0.05), Physical health (PH) (mean = 65.6 and 59.8, p < 0.001) and Mental health (MH) scores (mean = 68.5 and 64.9, p < 0.05) compared to women in mining community.

**Table 4:** SF36 among women in mining and agricultural communities using ANOVA

	**Functional status and Well-being**	**Mining community N = 145**		**Agricultural community N = 133**			
**Scale**	**SF36 Dimensions and items**	**Mean**	**SD**	**Mean**	**SD**	**F**	**P**
Physical health	1.Physical Functioning (PF)	50.1	26.1	53.7	21.1	1.5	0.22
	2.Physical Role limitations (RP)	57.8	44.6	79.9	35.3	20.8	0.00*
	3.Bodily Pain (BP)	69.3	25.1	73.2	19.9	2.0	0.15
	4.General health perceptions (GH)	61.5	15.3	59.6	13.4	1.1	0.29
	5.Vitality/energy/fatigue (VT)	60.4	15.0	61.8	13.7	0.7	0.42
Mental health	6.Social Functioning (SF)	65.9	23.5	72.7	20.0	6.7	0.01*
	7.Emotional–Role limitations (RE)	73.1	43.2	78.7	37.9	1.3	0.25
	8.Well-being or General Mental health (MH)	63.6	16.0	69.8	15.2	10.9	0.00*
	Physical health	59.8	15.8	65.6	13.7	10.7	0.00*
	Mental health	64.9	15.5	68.5	13.1	4.4	0.04*
	Total SF36	62.7	15.9	68.7	13.6	11.1	0.00*

* Significant at p < 0.05.

Physical health: subscales 1-5, Mental health: subscales 4-8.

The Physical role limitations, Social functioning, Well-being/General Mental Health subscales, Physical Health, Mental Health and Total SF36 scales were the most valid measures of SF36 among women in the agricultural community. These items showed higher mean scores than Bodily pain, General health perceptions or Vitality and Emotional-role limitations, suggesting that these problems did not lead to significant impairment in the faily activities or under-reporting of sensitive issues related to impairment. The mean scores in all the scales were higher among women in the agricultural community than among women in the mining community.

### Predictors of HRQOL

ANOVA was used to examine each of the predictors of Physical health, Mental health, and the total SF36 scores (Table 5). To reduce the number and size of the tables, all the categories of the predictors and the 8 sub-scales of the SF36 are not shown.

**Table 5:** Structural, health and psychosocial predictors of HRQOL among women in mining and agricultural communities using ANOVA

**Predictors**	**SF36**	**Mining community** **N = 145, p**	**Agricultural community** **N = 133, p**
Age (years)	Physical Health	0.02*	0.00*
	Mental Health	0.11	0.01*
	Total Health	0.03*	0.00*
Marital status	Physical Health	0.00*	0.09
	Mental Health	0.00*	0.01*
	Total Health	0.00*	0.03*
Years of stay in the area	Physical Health	0.11	0.03*
	Mental Health	0.37	0.12
	Total Health	0.15	0.04*
Educational status	Physical Health	0.34	0.00*
	Mental Health	0.55	0.00*
	Total Health	0.21	0.00*
Family type	Physical Health	0.84	0.2
	Mental Health	0.96	0.49
	Total Health	0.99	0.26
Economic status	Physical Health	0.00*	0.00*
	Mental Health	0.02*	0.00*
	Total Health	0.00*	0.00*
Domestic violence	Physical Health	0.00*	0.00*
	Mental Health	0.00*	0.00*
	Total Health	0.00*	0.00*
Enduring illness	Physical Health	0.00*	0.00*
	Mental Health	0.24	0.01*
	Total Health	0.03*	0.00*
Employment	Physical Health	0.13	0.01*
	Mental Health	0.3	0.01*
	Total Health	0.18	0.00*
Ownership asset	Physical Health	0.65	0.76
	Mental Health	0.04*	0.04*
	Total Health	0.3	0.31
Social coping	Physical Health	0.01*	0.43
	Mental Health	0.24	0.44
	Total Health	0.05*	0.89
Cognitive coping	Physical Health	0.10	0.03*
	Mental Health	0.01*	0.12
	Total Health	0.02*	0.05*
Emotional coping	Physical Health	0.01*	0.04*
	Mental Health	0.07	0.66
	Total Health	0.01*	0.22
Behavioural coping	Physical Health	0.00*	0.00*
	Mental Health	0.24	0.01*
	Total Health	0.03*	0.00*

* Significant at p < 0.05.

H01 There is no significant association between Physical and Mental Health means scores with Social Health Status and Coping Strategies among the women in mining as well as in the agricultural community.

There is a significant association between years of stay, education and employment status with the Physical Health, age, education, enduring illness, employment and behavioural coping with Mental Health and years of stay, education and employment with total health (SF36) and among women in the agricultural community (Table 5). A higher tendency to positive perception on HRQOL was observed with marital status, increased age, stay in the community, education, economic, employment status and ownership asset among women in the agricultural community. For SF36 a better perception of HRQOL is associated with absence of domestic violence and illness among these women. The existence of illness and domestic violence among women in the mining community promotes a less positive perception of HRQOL. The Physical Health component summary measure of the SF36 appeared to better explain differences in HRQOL among women in these communities.

A further General Linear Model (GLM) procedure was applied to investigate the relationship between PH and MH, interdependent variables with the predictors (independent variables) as seen in Tables 6 and 7.

**Table 6:** Overall model significance and tests of between-subjects effects using GLM

**Source**			**Mining community N = 145**			**Agricultural community N = 133**		
	**Health status**	**df**	**Mean Square**	**F**	**p.**	**Mean Square**	**F**	**p**
Corrected Model	Physical health	14	850.12	4.64	0*	697.58	5.49	0*
	Mental health		655.53	3.35	0	654.65	5.73	0*
Intercept	Physical health	1	684.6	3.74	0.06	3476.2	27.37	0*
	Mental health		2073.6	10.59	0*	5733.86	50.22	0*
Enduring illness	Physical health	1	1486.42	8.11	0.01*	564.12	4.44	0.04*
	Mental health		167.57	0.86	0.36	113.34	0.99	0.32
Age	Physical health	1	427.57	2.33	0.13	1144.72	9.01	0*
	Mental health		274.35	1.4	0.24	1244.24	10.9	0*
Marital status	Physical health	1	629.98	3.44	0.07	248.96	1.96	0.16
	Mental health		1523.03	7.78	0.01*	1035.69	9.07	0*
Years of stay	Physical health	1	92.16	0.5	0.48	153.87	1.21	0.27
	Mental health		156.68	0.8	0.37	207.04	1.81	0.18
Education	Physical health	1	204.76	1.12	0.29	182.34	1.44	0.23
	Mental health		80.75	0.41	0.52	60.52	0.53	0.47
Family type	Physical health	1	157.01	0.86	0.36	0.29	0	0.96
	Mental health		24.47	0.12	0.72	172.06	1.51	0.22
Employment	Physical health	1	853.15	4.66	0.03*	362.45	2.85	0.09
	Mental health		726.64	3.71	0.06	147.7	1.29	0.26
Ownership	Physical health	1	0.77	0	0.95	0.05	0	0.98
	Mental health		504.24	2.58	0.11	571.89	5.01	0.03*
Economic status	Physical health	1	326.1	1.78	0.18	797.2	6.28	0.01*
	Mental health		2.37	0.01	0.91	1171.78	10.26	0*
Domestic violence	Physical health	1	3066.98	16.74	0*	1021.71	8.05	0.01*
	Mental health		1929.47	9.86	0*	1240.13	10.86	0*
Emotional coping	Physical health	1	32.48	0.18	0.67	690.2	5.44	0.02*
	Mental health		26.65	0.14	0.71	70.7	0.62	0.43
Behavioural coping	Physical health	1	54.86	0.3	0.59	117.13	0.92	0.34
	Mental health		338.15	1.73	0.19	229.14	2.01	0.16
Social coping	Physical health	1	2650.92	14.47	0*	27	0.21	0.65
	Mental health		688.86	3.52	0.06	378.83	3.32	0.07
Cognitive coping	Physical health	1	622.34	3.4	0.07	719.18	5.66	0.02*
	Mental health		1172.41	5.99	0.02*	577.85	5.06	0.03*

# df = 1 *Significant at p < 0.05. Computed using alpha = 0.05.

Mining. Physical health: R2 = 0.333 (Adjusted R squared = 0.261), Mental health R2 = 0.265 (Adjusted R squared = 0.186). Agricultural. Physical health: R2 = 0.397 (Adjusted R squared = 0.324), Mental health: R2 = 0.407 (Adjusted R squared = 0.336).

**Table 7:** Combined effect of predictors on SF36 using Wilk’s Lambda Multivariate analysis of variants Tests (MANOVA/GLM)

**Wilks’ Lambda**	**Mining community N =145**			** Agriculture N =133**		
**Effect**	**Value**	**F**	**p**	**Value**	**F**	**p**
Intercept	0.92	5.46	0.01*	0.70	24.95	0.00*
Enduring illness	0.92	5.44	0.01*	0.96	2.44	0.09
Age	0.98	1.16	0.32	0.91	5.85	0.00*
Marital status	0.94	3.90	0.02*	0.92	5.01	0.01*
Years of stay	0.99	0.40	0.67	0.98	0.92	0.40
Education	0.99	0.57	0.57	0.99	0.73	0.49
Family type	0.99	0.54	0.59	0.97	1.57	0.21
Employment	0.96	2.44	0.09	0.98	1.42	0.25
Ownership	0.96	2.58	0.08	0.92	4.88	0.01*
Family economic status	0.97	1.68	0.19	0.92	5.15	0.01*
Domestic violence	0.89	8.33	0.00*	0.91	5.64	0.00*
Emotional coping	1.00	0.09	0.91	0.94	3.42	0.04*
Behavioural coping	0.98	1.04	0.36	0.98	1.00	0.37
Social coping	0.89	8.04	0.00*	0.93	4.65	0.01*
Coginitive coping	0.96	2.97	0.05*	0.95	3.13	0.05*

*Significant at p < 0.05. Design: Intercept + enduring illness (present**, absent) + age (less than 30, 30–39 and more than 40 years**) + marital status (living with husband, separated/divorced**) + years of stay (less than 10, 10–20, more than 20 years**) + education (no schooling, elementary, secondary, vocational**) + family type (joint**, nuclear) + family economic status(good**, moderate, poor) + domestic violence(wife beating**, no assault) + emotional coping (present**, absent) + behavioural coping(present**, absent) + social coping(present**, absent) + coginitive coping(present**, absent). **Reference groups used in regression.

From the ANOVA tests (Table 5) factors such as younger age, marriage, shorter duration of stay in the community, employment, education, economic status, domestic violence, enduring illness, house ownership, social, cognitive, emotional and behavioral coping among women significantly predicted better Physical Health among women both in the agricultural and mining communities. Age, marriage, economic status, education, employment, ownership, domestic violence and illness significantly predicted better Mental health among women across both communities. Younger age, marital status, economic status, domestic violence, enduring illness, cognitive and behavioral coping among these women significantly predicted better SF36 scores. In addition, years of stay, education and work influenced Physical Health; age, education, enduring illness, work and behavioral coping led to better Mental Health and the SF36 scores was also influenced by years of stay, education and work among women in the agricultural community. Social coping improved Physical Health, cognitive coping affected Mental Health, and social and emotional coping predicted higher SF36 scores among women in the mining community.

The MANOVA models were used with all the variables in the ANOVA tests as predictors of PH and MH to test the combined effects of all independent variables together (Table 6). The MANOVA results are explained with the test of overall model significance and the test of overall individual effects of predictors. 39% (33%) and 40% (26%) of the variance in Physical and Mental Health among women was found in agricultural and mining communities respectively and was predicted by the structural, health and psychosocial coping variables. The Physical and Mental Health components were strongly correlated with the demographic (marital, employment status), cultural (domestic violence), health (enduring illness) and coping (social and cognitive) predictors. Though the R^2^ values were low, these results were used as indicative support of the relationship among the predictors. Physical and Mental Health have had a significant positive effect on HRQOL.

The test of overall model significance showed that the model is significant for each dependent variable (PH and MH) in the mining and the agricultural community. The “Tests of Between Subjects Effects” applies F test of significance to the relation of each covariate (age, illness, coping etc.) to each of the dependent variables (PH and MH). From the MANOVA test (Table 6) age, marriage, employment, economic status, domestic violence, social and cognitive coping significantly predicted Physical Health. Age, marital status, employment, economic status, social and cognitive coping predicted Mental Health among women across both the communities.

Women in the agricultural community had better Physical Health related to age, economic status, emotional and cognitive coping and better Mental Health related to age, ownership and economic status. Women in the mining community had better Physical Health predicted by marital status and social coping and Mental Health predicted by employment. The combined effect of predictors on PH and MH using Wilk’s Lambda Multivariate Tests (Table 7) show illness, domestic violence, employment, social and cognitive coping among women in the mining and agricultural communities were significant predictors with this test (Table 7). Marriage and employment is the only other variable significant among women in the mining community whereas age, economic status and emotional coping emerged as other significant predictors among women in agricultural community. Marital status, domestic violence, social and cognitive coping were found to be significant predictors of the SF36 among women across both communities (Tables 6 and 7). In addition, age, economic status and emotional coping were significant predictors among women in the agricultural community.

## Discussion

We could have employed an ethnographic qualitative research approach to assess the coping strategies used among women at different points in time to uncover explanations related to the why and how of the perceived health status. Future research should consider the use of a prospective longitudinal design to gather data for a better understanding of how women adapt over time with stress in economically stressed communities and how this affects various dimensions of their health-related quality of life.

Women in the agricultural community scored higher on all dimensions of Physical functioning (PF), Physical role limitations (RP), Bodily pain (BP), Vitality/energy/fatigue, General health (GH), Mental health (MH), Social functioning (SF) and Emotional role limitations (ER) compared with women in mining. The perception of decreased illness and domestic violence is a significant predictor of health status among women in agriculture. They had higher education, employment, ownership and more coping strategies which paralleled a marked increase in PH, MH and SF36 scores among these women. These women perceived more role functions, a better self-concept and interdependence and felt the need to return to gainful employment and family roles. This shows a reciprocal relationship between physical role limitations, social functioning and general mental health among women that supports the higher order components (Physical health, Mental health and Total health). This stipulates that their good physical health presupposes good mental health and vice versa among women in the agricultural community. Coping strategies were significantly higher and positively related to the HRQOL among women in the agricultural community. Higher capacity for coping are positively associated with HRQOL [60-63] which showed higher capacity for coping and positively associated with quality of life [64].

Women in the mining community reported higher levels of eye, upper and lower respiratory and psychological illness as well as higher percentage of domestic violence. This can be explained by less exposure to education, employment, experiencing stressful events (environmental stress like dust and air pollution) and lack of power, control and autonomy [65,66]. Stress and stressful life events are related to a number of disorders, both psychological and physical, among women [27,28,67,68]. Clinical variables (symptoms, demographic, biological, physiological factors) are linked to and are predictive of HRQOL [69,70]. The connection between domestic violence and illness is a significant predictor of poor Physical and Mental Health.

Age, economic status, years of stay, domestic violence and illness are significantly associated with quality of life and reproductive health index among women in the mining community [51]. Age, marital duration, poverty, gender inequities, husband’s employment [17] and gender-based power dynamics [71,72] emerged as the risk factors of victimization and the perpetration of all types of domestic violence. Educated [73] and regularly employed women are typically more autonomous and possess the resources and skills necessary to better recognize and terminate a potentially abusive relationships and were less likely to report ever having experienced physical domestic violence than unemployed women [71].

The SF36 correlates were high with decreased illness, age, marital status, ownership asset, domestic violence, emotional, social and cognitive coping among women in the agricultural community. In our study, role functions, social valuing of role expectations and mental health has been identified as motivators in the development of good HRQOL among women in the agricultural community. Women’s health differs in various communities because they also react in different ways to predictors that determine health. Health status in stressed communities (mining) is lower than for non-stressed communities (agricultural), and limited educational and employment can further jeopardise health status. The socio-cultural environment exerts a constraining impact on coping and the SF36 among women in mining communities, leading to a stressful life; while limited educational and employment opportunities may compound to aggravate Physical and Mental Health.

## Conclusion

In our study Physical and Mental Health have significantly positive and direct influence on the HRQOL among women. HRQOL is determined by complex layers of intertwined forces, with structural (age, marital status, economic, family type, ownership), health (illness, domestic violence) and psychosocial (social, emotional, cognitive and behavioural coping strategies) predictors of Physical and Mental Health among women in the agricultural and mining communities. Psychosocial resources, health and stressful events are rooted in the social structural context of women’s lives. In addition years of stay, education and employment are significant predictors among women in the agricultural community. Cognitive-perceptual factors and self–efficacy were significant predictors of health promoting lifestyle and HRQOL [74].

The differential pattern of responses related to cultural and health (domestic violence, illness), socio-economic factors (education, employment, economy) and coping strategies (social, emotional, behavioural and cognitive) signifies exposure to focal (unsupportive relationships, violence), contextual (job stress, financial instability) or residual (environmental stress) stimuli, are positively associated with the SF36 dimensions among women in the mining and agricultural communities. These stimuli have both direct and mediating responses on HRQOL which explain the differences in perceived health among women across both communities. This perspective helps to illuminate those aspects of the adaptive system that contribute to a differential exposure to stimuli and to the coping strategies (resources) that women mobilize against stimuli or problems [2,5,75].

Coping strategies or resources (cognitive and emotional) vary in their benefits and are positively related to better HRQOL among women in various communities. Women’s coping strategy (psychosocial predictors) is an important factor which affects health outcomes and influences HRQOL. The SF36 dimensions (HRQOL) were found to reflect the health status of women and were also significantly associated with coping with stress which plays a key role in women’s physical and mental health. Coping style has a strong association with life satisfaction and well-being [76]. Coping strategies and resources play an important role in determining adaptation to health related quality of life, but their effects have been stronger for women in the agricultural compared with the mining community. Women apply specific coping strategies when encountering stimuli, for example cognitive coping attempts to alter and manage the stressor, whereas emotional/social coping attempts to regulate the emotional response to the stressor. Our results contribute to an increase in awareness of coping as important indicators of successful adaptation. Cognitive coping improves health status and was positively related to better quality of life among women in the agricultural community. Women who are not able to maintain physiologic-physical needs (polluted environmental, mental stress), poor self-concept, inadequate role function as a family member, spouse, interdependence (poor self-esteem, poor communication), find it difficult to maintain daily activities and interaction with significant others such as family members. Women in the agricultural communities are at a greater advantage with better biopsychosocial, coping processes and adaptive modes leading to effective responses and adaptive behaviours. This study shows that structural, health and psychosocial predictors determine HRQOL among women in both the communities.

### Implications for nursing

SF-36 is a diagnostic tool for nurses to assess the functional health patterns and perceived health needs of high-risk women subgroups in vulnerable populations and settings. Self-assessment of health is important to facilitate more individualized nursing care to promote HRQOL among women. Identification of stimuli and coping strategies that are predictive of health status are critical to the development of coping strategies in these populations of women. Nurses can address the importance of valuing role functions among women and this information can be used for designing appropriate nursing interventions. Thereby women can be empowered to make informed decisions and to act on them. This ability to discriminate across vulnerable populations means that nurses can use SF36 health scores to better understand functional status and the health care needs of at-risk women sub-groups. This will enable health policy makers to evaluate the health status and outcomes among women for enhance policy making towards optimum resource management and provision of safe environment.

## Appendix A

### Definition of terms

Focal stimuli are those most immediately confronting the person.

Contextual-all other stimuli present that are affecting the situation and Residual- those stimuli whose effect on the situation are unclear.

Adaptation: the process and outcome whereby thinking and feeling persons, as individuals and in groups, use conscious awareness and choice to create human and environmental integration

Adaptive Responses: responses that promotes integrity in terms of the goals of the human system, that is, survival, growth, reproduction, mastery, and personal and environmental transformation

Ineffective Responses: responses that do not contribute to integrity in terms of the goals of the human system

An adaptive system with coping processes, cognator and regulator subsystems acting to maintain adaptation in the four adaptive modes: physiologic-physical, self-concept-group identity, role function, and interdependence.

Regulators regulate four adaptive modes through neuro chemical or endocrine activities. Cognator are the control subsystem. Roys view the regulators and cognator as method of coping. When all six sub systems function well adaptative behaviour will occur otherwise ineffective response or maladaptation will occurs.

### Adaptive modes

Physiologic-physical. Physical and chemical processes involved in the function and activities of living organisms; the underlying need is physiologic integrity as seen in the degree of wholeness achieved through adaptation to change in needs. Five needs-oxygenation, nutrition, elimination, activity and rest, protection. For complex processes-senses; fluid, electrolyte, and acid–base balance; neurologic function; endocrine function

Self-concept-group identity. Focuses on psychological and spiritual integrity and sense of unity, meaning, and purposefulness in the universe. Need is psychic and spiritual integrity so that one can be or exist with a sense of unity, meaning, and purposefulness in the universe

Role function. Roles that individuals occupy in society, fulfilling the need for social integrity. It is knowing who one is in relation to others. Need is social integrity; knowing who one is in relation to others so one can acct; role set is the complex of positions individual holds; involves role development, instrumental and expressive behaviors, and role taking process

Interdependence: The close relationships of people and their purpose, structure and development individually and in groups and the adaptation potential of these groups. Need is to achieve relational integrity using process of affectional adequacy, i.e., the giving and receiving of love, respect, and value through effective relations and communication.

### Research highlights

○ Physical and Emotional Role Limitations are motivators to promote coping among women for health related quality of life.

○ Age, marital status, economic status, family type, ownership, illness and domestic violence are predictors among women.

○ Cognitive, emotional and behavioural coping strategies influence women’s Physical and Mental health and health-related quality of life.

○ SF-36 Health Survey should be used to assess and plan nursing interventions for improving health-related quality of life.

## Abbreviations

BP: Bodily pain; GH: General health; HRQOL: Health-related quality of life; MCS: Mental health component summary; MH: Mental health; PCS: Physical component summary; PF: Physical functioning; RE: Role emotional; RP: Role physical; SF: Social functioning; SF-36: Short form 36-item health survey; VT: Vitality; PH: Physical health; QOL: Quality of life.

## Competing interests

The authors declare that they have no potential competing interests.

## Authors’ contributions

All authors meet the criteria for authorship, have designed, provided analysis and interpreted the data, drafted, revised and approved the final article and those entitled to authorship are listed as authors. MSD conceived of the study conception and design, data collection/acquisition, and provided analysis and interpretation. KSN participated in the data collection/acquisition and provided analysis and interpretation. GS carried out the study conception and design. All the authors drafted the manuscript, provided critical revision of manuscript for important intellectual content and final approval of version to be submitted. MSD-Melba Sheila D’Souza; KSN- Karkada Subrahmanya Nairy; GS-Ganesha Somayaji.
